# Analysis of coffee bean extracts by use of ultra-performance liquid chromatography coupled to quadrupole time-of-flight mass spectrometry

**DOI:** 10.1016/j.mex.2014.10.006

**Published:** 2014-11-03

**Authors:** Daniel James O’Driscoll

**Affiliations:** Alimentary Pharmabiotic Centre, Biosciences Institute, University College Cork, Cork, Ireland

**Keywords:** UPLC, Q-TOF/MS, Caffeine, Mass spectrometry, Phenolic analysis, Waters

## Abstract

The number of flavour chemicals identified in coffee has reached over 1000 [1], [2]. Coffee is one of the world's most popular beverages [3], highly studied for its health-related properties [4], [5], [6]. Studies on coffee associated with human health have focused on the negative aspects, such as the toxicity of caffeine [7], [8]. Complex chemistry happens during coffee roasting and according to the literature, a number of compounds have been detected and quantified in coffee beans samples by UPLC–Q-TOF/MS [9], [10], [11], [12]. The following method offers a simple approach for the qualitative and quantitative analysis of coffee bean extracts using a Waters Acquity G2 UPLC–Q-TOF/MS instrument adapted from the method by Kenny et al., [12]. The following modifications were made:

• The method by Kenny et al. was developed on a triple quadrupole mass spectrometer, the below method was developed on a Q-TOF MS.
• A combination of utilising both base peak index and mass extraction at 0.05 Da allows for a sensitive, quantitative technique amidst poor background noise and poor separation with high mass accuracy (<5 ppm).
• By use of MS^E^ centroid experiment, greater mass spectral information for metabolite profiling could be obtained.

## Graphical abstract

**Figure:**
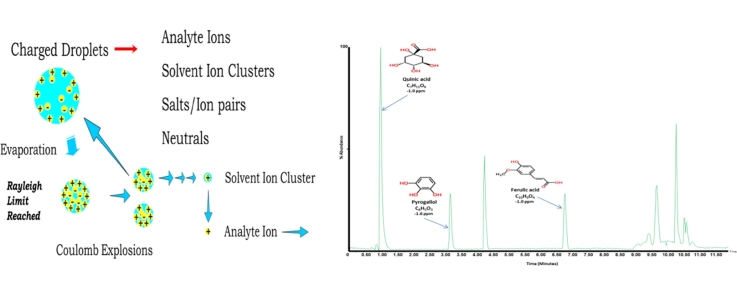

## Method details

### Liquid chromatography mass spectrometry analysis

The analysis was performed using a Waters Acquity G2 Q-TOF LC–MS instrument. This system is composed of a Waters Acquity UPLC system coupled to a quadrupole time-of flight mass spectrometer. The samples were eluted using a Titan C18 HPLC analytical column (100 mm × 2.1 mm, 1.9 μm) and preceded by a Titan C18 guard cartridge (5 mm × 2.1 mm, 1.9 μm) with the column set to 35 °C. All samples were kept refrigerated to 4 °C in the UPLC autosampler and a 10 μL injection volume was used with a total flow rate of 0.3 mL/min over a total run time of 12 min. All solvents used were LC–MS grade and ultra-pure 18.2 MΩ water was used for each step. Mobile phase A consisted of water + 0.1% formic acid while mobile phase B was acetonitrile + 0.1% formic acid. The following tables contain the gradient details for list of compounds analysed (Table 1, Table 2):

Mass spectrometry detection was conducted through electrospray ionisation using an ms^E^ centroid experiment in both positive and negative mode and screened in the *m*/*z* scan range of 50–2000 Da (Table 3) with the analyser set to resolution mode at FWHM. Scanning conditions were set to 1 scan every 0.7 s. Collision energy was set for two functions, function one at low energy with no collision energy applied and function two at high energy using a collision energy ramp from 20 to 75 eV. In negative mode the following MS tune file settings were used: Capillary voltage 3.00 kV, sampling cone 40 V, extraction cone 4.0 V, source temperature 120 °C, desolvation temperature 450 °C, desolvation gas flow 800 L/h, cone gas flow 50 L/h. In positive mode, the following MS tune file settings were used: capillary voltage 3.00 kV, sampling cone 30 V, extraction cone 2.0 V, source temperature 120 °C, desolvation temperature 450 °C, desolvation gas flow 800 L/h, cone gas flow 50 L/h. The accurate mass of the instrument was initially calibrated through direct infusion of a sodium iodide calibrant solution prior to sample analysis. In addition, leucine enkephalin (Leuenk) lockmass solution (2 ng/uL) was infused at 5 μL/min in parallel to the mobile phase flow, scanned and automatically corrected to verify exact mass which ensured high mass accuracy (<5 ppm) throughout the scan range over the course of the submitted sequence. Masslynx v4.1 software was used to control the instrument and also analyse the data.

### Sample extraction

Coffee beans (green and roasted) were frozen with liquid nitrogen and ground with a mill. Ground coffee samples (2 g) were extracted with LC grade water at 92 °C (25 mL) then stirred for 6 min at 70–80 °C and placed on ice immediately after in order to cool down rapidly. The samples were centrifuge at 21,481 × *g* for 2 min. After centrifugation the extracts were filtered through a 0.2 μm PVDF membrane. Extracts were poured into 1.5 mL vials and sealed. All other remaining samples and extracts were kept in the freezer at −20 °C.

### Stock solution preparation

Two stock solutions were prepared, these included methanol and water depending on the solubility of the compound. All standards were prepared between 1 and 8 mg to a final volume of 10 mL. The methanol stock solution consisted of caffeine, 5-caffeoylquinic acid, vitamin B3, caffeic acid, catechol and 1,2,4-benzenetriol. While the water stock solution consisted of trigonelline hydrochloride, quinic acid, ferulic acid and pyrogallol.

### Quantification

Quantification was performed by generation of suitably linear curves for each of the analysed standards (Table 4). All standard curves were created in Microsoft Excel, 2010. For the purpose of this method it was deemed necessary to determine only limit of detection (LOD), limit of quantitation (LOQ) and finally linearity over the range to obtain suitable *R*^2^ values. In order to establish LOD and LOQ values, each analyte was determined as concentrations equivalents to three times and 10 times the signal-to-noise ratio of the compounds of interest in the lowest concentration of the calibration curve prepared using the green bean extract. The signal-to-noise ratios were calculated using Waters MassLynx software version 4.1

### Base peak index (BPI) full scan MS chromatographic profiles

**Fig. 1:**
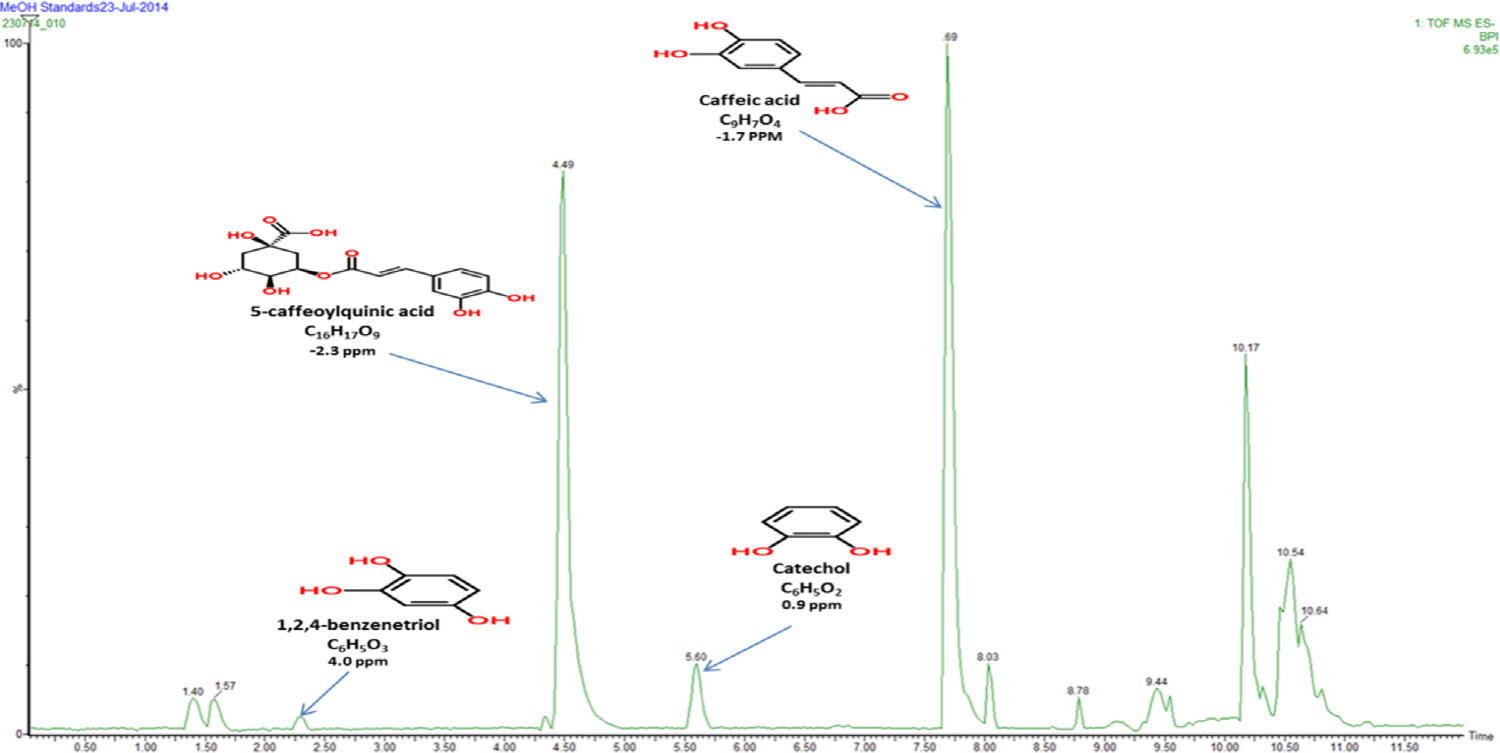
Full scan MS (negative mode) chromatogram conducted in the mass to charge (*m*/*z*) range between 50 and 2000 Da displaying elemental composition and error ppm reconstituted in MeOH.

**Fig. 2:**
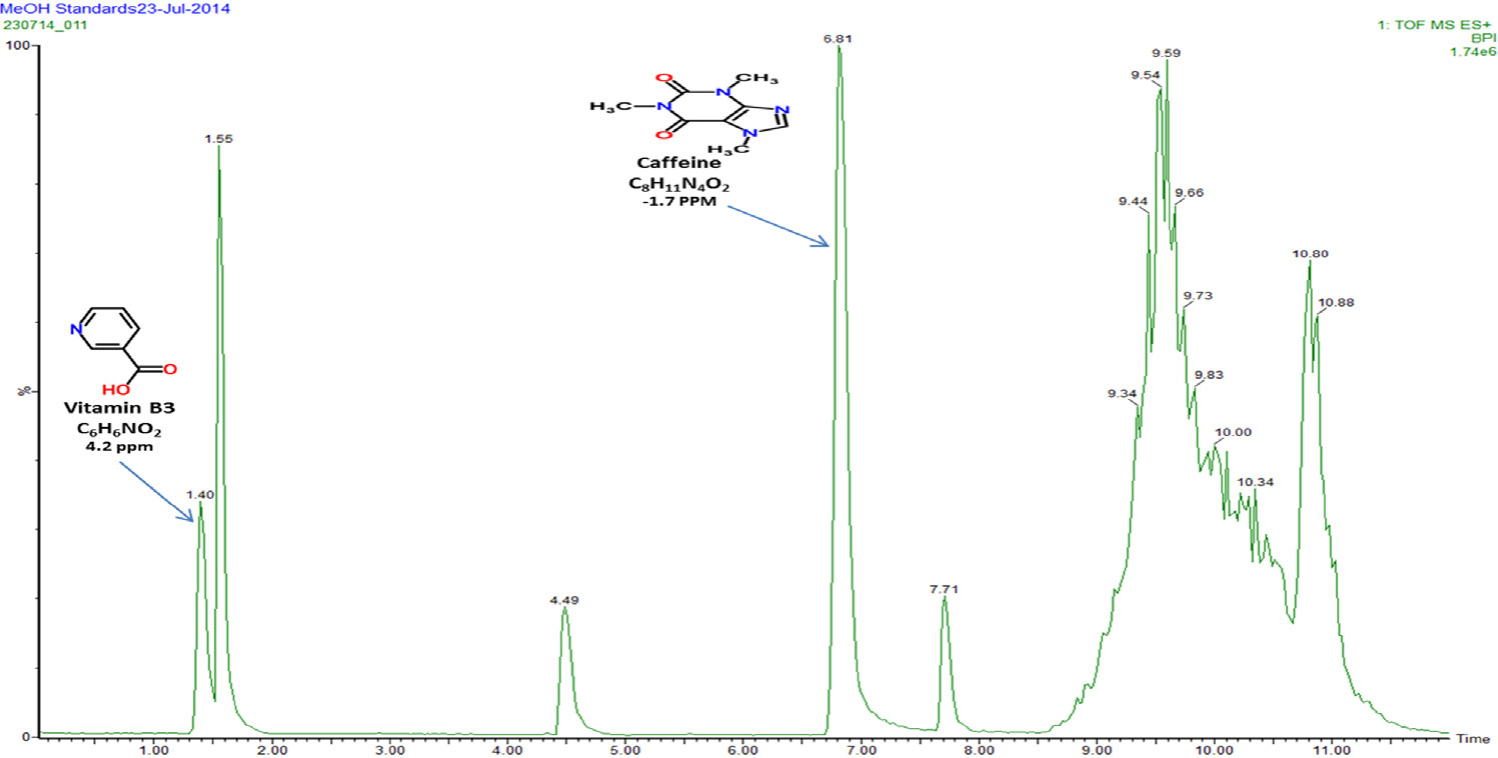
Full scan MS (positive mode) chromatogram conducted in the mass to charge (*m*/*z*) range between 50 and 2000 Da displaying elemental composition and error ppm reconstituted in MeOH.

**Fig. 3:**
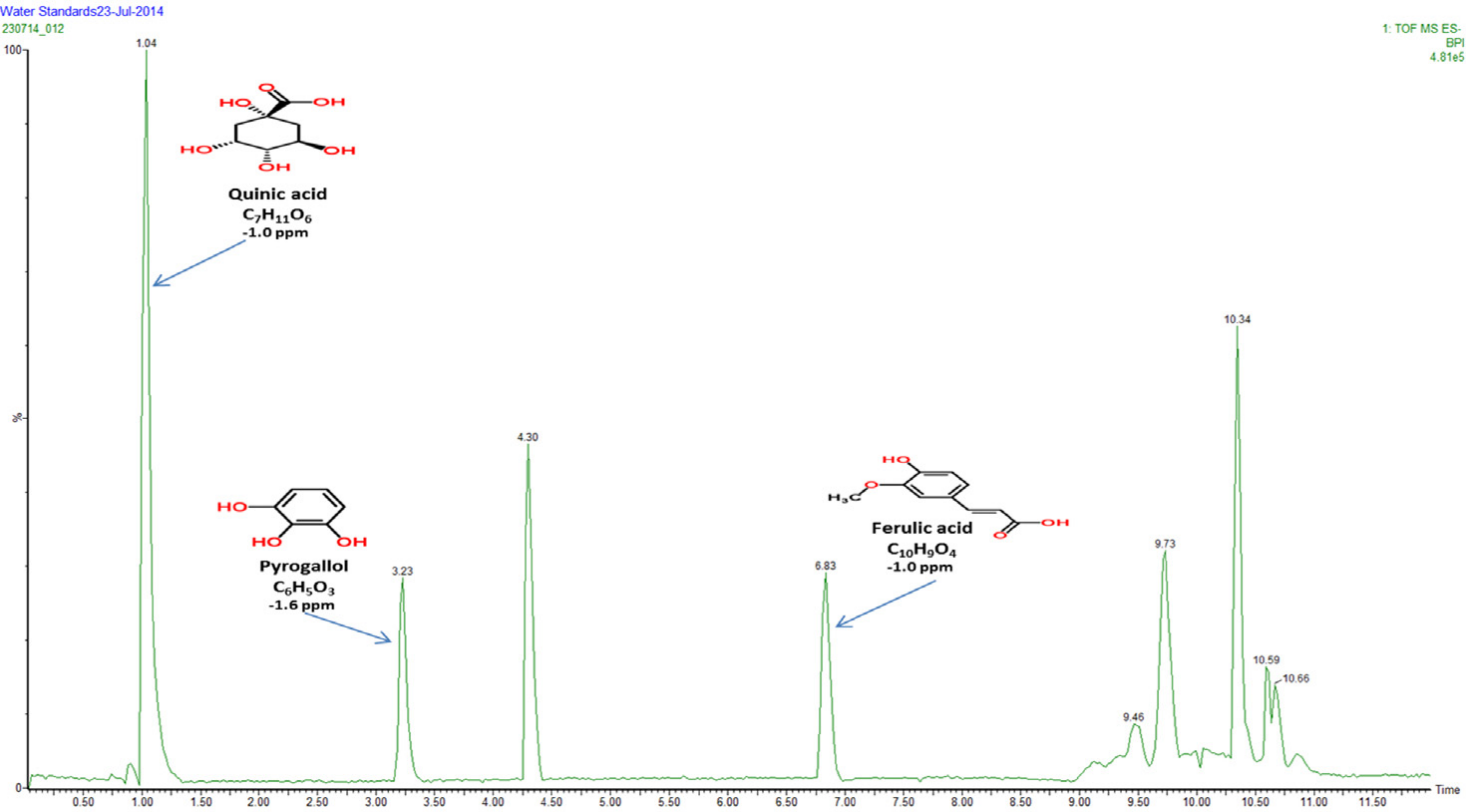
Full scan MS (negative mode) chromatogram conducted in the mass to charge (*m*/*z*) range between 50 and 2000 Da displaying elemental composition and error ppm reconstituted in water.

**Fig. 4:**
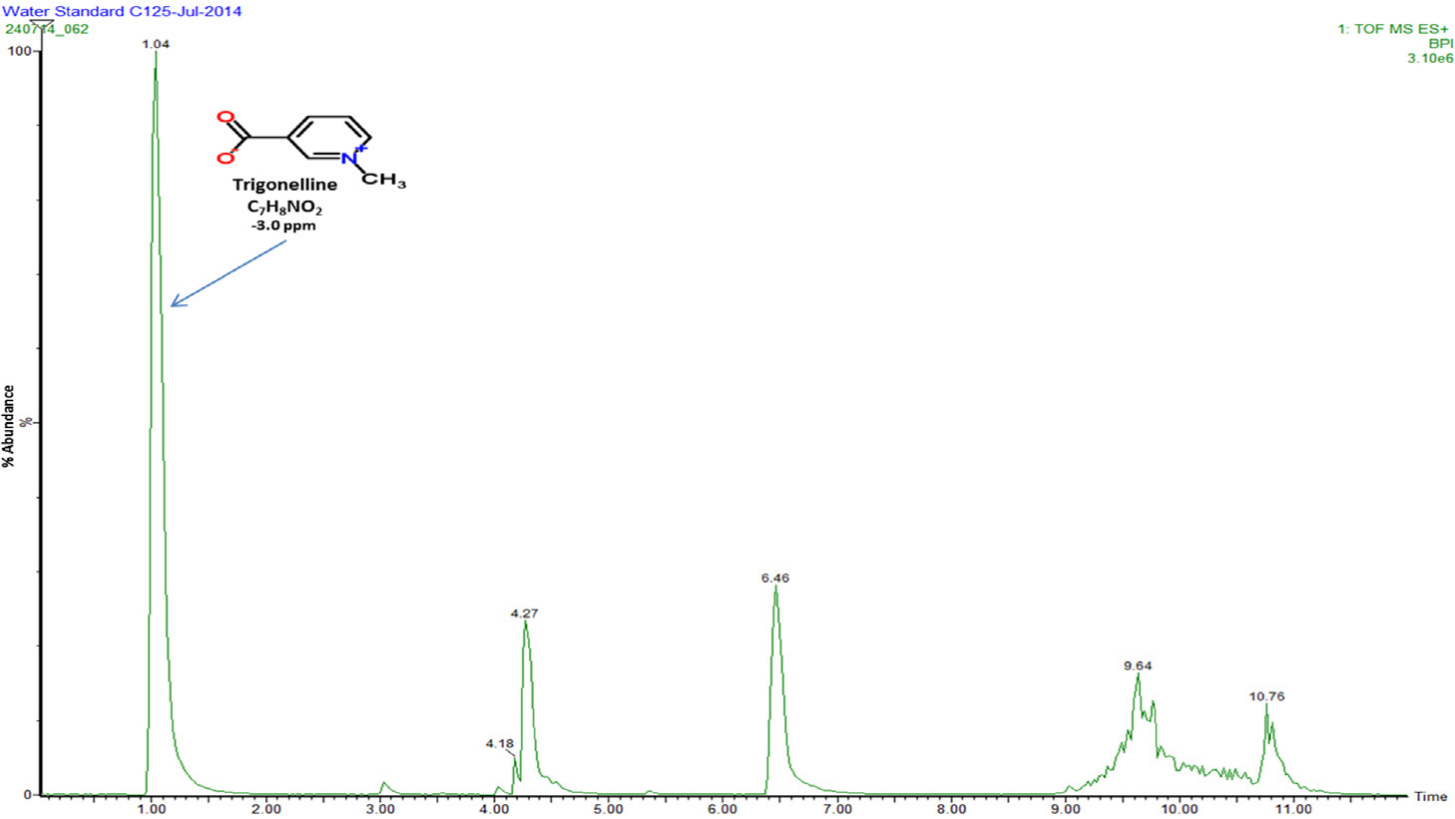
Full scan MS (positive mode) chromatogram conducted in the mass to charge (*m*/*z*) range between 50 and 2000 Da displaying elemental composition and error ppm reconstituted in water.

### Discussion of figures

Fig. 1, Fig. 2 represent total ion count chromatograms generated from the analysis of methanol in both positive and negative mode mass spectrometry while Fig. 3, Fig. 4 were analysed in water. From the peaks obtained it can be seen that some compounds ionise in both positive and negative polarity, labelled peaks represent best ionisation. In Fig. 1, peak at retention time 1.40 represents vitamin B3, in Fig. 2 the peaks at retention time 4.49 and 7.71 represent both 5-caffeoylquinic acid and caffeic acid, respectively. For confidentiality reasons, peak at retention time 4.30 in Fig. 3, peak at retention time 1.56 in Fig. 1, Fig. 2 and finally peaks at retention times 4.27 and 6.46 cannot be disclosed.

## Figures and Tables

**Table 1:** Gradient used for separation and identification of standards reconstituted in water include quinic acid, ferulic acid, pyrogallol, and trigonelline hydrochloride.

Time (min)	Flow rate	%A	%B
Initial	0.3	98.0	2.0
1	0.3	98.0	2.0
2	0.3	90.0	10.0
3	0.3	80.0	20.0
6	0.3	80.0	20.0
7.5	0.3	65.0	35.0
8.5	0.3	10.0	90.0
9.5	0.3	10.0	90.0
12	0.3	98.0	2.0

**Table 2:** Gradient used for separation and identification of standards reconstituted in MeOH include caffeine, 5-caffeoylquinic acid, vitamin B3, caffeic acid, catechol, and 1,2,4-benzentriol.

Time (min)	Flow rate	%A	%B
Initial	0.3	98.0	2.0
1	0.3	98.0	2.0
2	0.3	90.0	10.0
3	0.3	90.0	10.0
6	0.3	90.0	10.0
7.5	0.3	50.0	50.0
8.5	0.3	10.0	90.0
9.5	0.3	10.0	90.0
12	0.3	98.0	2.0

**Table 3:** List of compounds (standards) qualitatively and quantitatively analysed in both positive and negative mode ionisation.

Molecular formula	Monoisotopic mass (Da)	Compounds	Solvent solubility	Polarity (+/−)	% Error ppm
C_8_H_10_N_4_O_2_	194.080383	Caffeine	MeOH	+	3.1
C_7_H_7_NO_2_	137.047684	Trigonelline hydrochloride	Water	+	5.0
C_16_H_18_O_9_	354.095093	5-Caffeoylquinic acid	MeOH	−	−2.3
C_7_H_12_O_6_	192.063385	Quinic acid	Water	−	−1.0
C_6_H_5_NO_2_	123.032028	Vitamin B3	MeOH	+	4.2
C_10_H_10_O_4_	194.057907	Ferulic acid	Water	−	−1.0
C_9_H_8_O_4_	180.042252	Caffeic acid	MeOH	−	−1.7
C_6_H_6_O_2_	110.036781	Catechol	MeOH	−	0.9
C_6_H_6_O_3_	126.031693	Pyrogallol	Water	−	−1.6
C_6_H_6_O_3_	126.031693	1,2,4-Benzenetriol	MeOH	−	4.0

**Table 4:** *R*^2^ values for calibration curves (n=8) generated from Waters Acquity G2 UPLC–Q-TOF/MS instrument.

Compounds	Concentration range (mg/mL)	LOD (mg/mL)	LOQ (mg/mL)	R^2^	Sample Green Bean (mg/mL)
Caffeine	0.00036–0.00359	0.0000119	0.0000396	0.9967	0.00040
Trigonelline hydrochloride	0.00047–0.00367	0.0000364	0.0001215	0.9954	0.00202
5-Caffeoylquinic acid	0.00039–0.0028	0.0000185	0.0000637	0.9961	nd
Quinic acid	0.00026–0.00203	0.000020	0.000080	0.9984	0.00143
Vitamin B3	0.00042–0.00328	0.0000185	0.0000617	0.9981	0.0009
Ferulic acid	0.00038–0.00297	0.000020	0.000080	0.9943	0.00100
Caffeic acid	0.00043–0.00336	0.000020	0.000080	0.9974	0.00273
Catechol	0.00015–0.00117	0.000020	0.000080	0.9982	0.00066
Pyrogallol	0.00021–0.00164	0.000020	0.000080	0.9976	0.0003
1,2,4-Benzenetriol	0.0001–0.00781	0.000020	0.000080	0.9966	0.00134
